# Applied Cliplets-based half-dynamic videos as intervention learning materials to attract the attention of adolescents with autism spectrum disorder to improve their perceptions and judgments of the facial expressions and emotions of others

**DOI:** 10.1186/s40064-016-2884-z

**Published:** 2016-07-29

**Authors:** I-Jui Lee, Chien-Hsu Chen, Ling-Yi Lin

**Affiliations:** 1 Ergonomics and Interaction Design Lab, Department of Industrial Design, National Cheng Kung University, No.1 University Rd., East District, Tainan, Taiwan; 2 Department of Occupational Therapy, College of Medicine, National Cheng Kung University, No.1 University Rd., East District, Tainan, Taiwan

**Keywords:** Half-static and half-dynamic video, Selective attention, Facial expressions, Nonverbal social cues

## Abstract

**Background:**

Autism spectrum disorders (ASD) are characterized by a reduced ability to understand the emotional expressions on other people’s faces. Increasing evidence indicates that children with ASD might not recognize or understand crucial nonverbal behaviors, which likely causes them to ignore nonverbal gestures and social cues, like facial expressions, that usually aid social interaction.

**Objective:**

In this study, we used software technology to create half-static and dynamic video materials to teach adolescents with ASD how to become aware of six basic facial expressions observed in real situations.

**Methods:**

This intervention system provides a half-way point via a dynamic video of a specific element within a static-surrounding frame to strengthen the ability of the six adolescents with ASD to attract their attention on the relevant dynamic facial expressions and ignore irrelevant ones.

**Results:**

Using a multiple baseline design across participants, we found that the intervention learning system provided a simple yet effective way for adolescents with ASD to attract their attention on the nonverbal facial cues; the intervention helped them better understand and judge others’ facial emotions.

**Conclusion:**

We conclude that the limited amount of information with structured and specific close-up visual social cues helped the participants improve judgments of the emotional meaning of the facial expressions of others.

## Background

ASD is a neurodevelopmental condition defined by impairments across the areas of reciprocal social interaction problems with verbal and nonverbal communication and with repetitive and stereotyped behaviors (Boelte and Hallmayer 2013). People with ASD have a range of cognitive and affective difficulties recognizing feelings in themselves and others (Lacava et al. 2007). Although people with high-functioning autism (HFA) may perform better in recognizing basic emotions, they have difficulty understanding more complex emotions (Bauminger 2004; Capps et al. 1992; Hillier and Allinson 2002). In addition, among the most characteristic early symptoms of ASD are atypical eye contact and joint attention, which profoundly impair the development of social-communication skills (Senju and Johnson 2009). Several eye-tracking studies of young children with ASD illustrate an emerging consensus that detailed characterizations at the level of eye movements in response to fixating and tracking visual stimuli are important (Falck-Ytter et al. 2013). Children with ASD typically have behavioral difficulties that suggest problems with visual attention; it is unclear whether this attention deficit causes the other symptoms of ASD or is a consequence of the disorder (Koldewyn et al. 2013). Research (Durham University News 2013) has indicated that children with ASD might be missing crucial nonverbal indicating behaviors, which likely causes children with ASD to not be able to recognize or understand nonverbal gestures and social cues, like facial expressions, that usually aid social interaction. Missing these cues generally has a negative effect on their social interaction skills and the flow of their communication (Mundy et al. 1986) because people with ASD cannot interpret other people’s facial expressions and emotional states, or understand the intentions and internal activities of others (Krasny et al. 2003). They also cannot respond with appropriate gestures, postures, or proximity (Ryan and Ni Charragain 2010)—a defect that researchers have called the Theory of Mind ability: the skill to view things from other people’s perspective and to understand the mental states of others (Smith 2006)—i.e., the ability to empathize (Baron-Cohen and Belmonte 2005; Baron-Cohen et al. (1985). Therefore, children with ASD, who normally pay more attention to inanimate objects than to faces, need to be taught specific verbal and nonverbal indicating behaviors involved in social interactions, and must learn to pay attention to the faces of people they meet and talk to, in order to understand social emotional behavior (Martins and Harris 2006; McPartland et al. 2011).

### Interventions to improve specific perception judgments of facial expressions

Blum-Dimaya et al. (2010) reported that using facial pictures and video training taught children with ASD to develop social communication skills and to focus on the specific visual representation and facial cues to judge others’ emotions. Some researchers also believe that people with ASD have an impaired ability to understand complex emotional and social information from facial stimuli (Baron-Cohen et al. 2001). Facial expressions are a key determinate of nonverbal cues in social development and the ability to interact with others (Back et al. 2007; Baron-Cohen et al. 1997). Moreover, understanding social situations requires paying attention to other people and to the subtle social cues they generate. However, people with ASD lack these abilities, especially an intuitive awareness and ability to judge the facially expressed emotions of others.

Golan et al. (2010), using the Transporters DVD (http://www.thetransporters.com/) as a learning tool to attract the attention of children with ASD, focused on the expressions of animated human faces on toy trains, buses, and other vehicles (transporters) that are characters in a video story which illustrates, names, and describes emotions in some common social situations. They reported that their participants’ ability to judge others’ emotions significantly improved. Thus, evidence shows that video-based interventions (VBIs) such as Video Modeling (VM) have been therapeutically effective for teaching functional, social, and behavioral skills to children with ASD (Ayres and Langone 2005; Bellini and Akullian 2007; Corbett and Abdullah 2005). Although VBIs are advantageous for promoting the motivation of children with ASDs to learn, the children still have difficulty dynamically adjusting the size of their attentional focus and switching the locus of their attention (Elsabbagh et al. 2009; Facoetti et al. 2008; Ibanez et al. 2008; Kikuchi et al. 2011; Landry and Bryson 2004; van der Geest et al. 2001), especially in patterns that include dynamic, repetitive, or social stimuli. It has been suggested (Koldewyn et al. 2013), however, that decreased multiple object tracking (MOT) performance is not due to deficits in dynamic attention but to a diminished capacity to select and maintain attention on multiple targets. Therefore, several problems that may occur, such as a lack of progress, could be due to a lack of reinforcement of sustained attention, poor video content, or a lack of prerequisites (Sigafoos et al. 2007).

### Cliplets-based half-dynamic video as intervention materials

In this study, we reduced multiple targets in specific social areas to create half-static and half-dynamic video called Cliplets-based video (CBV) for our intervention-based learning materials. We hypothesize that CBVs require less attention and generate less stress than normal dynamic video (DV) because they have fewer visual channels requiring attention due to the freezing of certain unimportant parts of the DV so as not to distract watchers. Be that as it may, the ignored parts can also support their situational awareness so as to more accurately experience the status of the scenario. In the fundamental theory and practice of ergonomics, when people are required to pay attention to more signal channels, the person’s stress and mental load will increase because the short term sensory store is limited in its ability to process information, and so handling large amounts of information requires the selective focus of attention (Dzubak 2008; Valkenburg 2011). Theories differ as to whether humans employ a single resource or multiple resources to manage the use of this limited capability (Nemeth 2004). Attention is a limited resource, and people have a fixed amount that must be allocated according to need (Scalf et al. 2013). To use a popular analogy, attention is like a bucket of water. People draw upon it as needed, but every dipper full and every teaspoon full leaves less for other purposes (Marc Green 2013). However, total attentional capacity varies according to circumstance (Swallow and Jiang 2013) therefore, the CBV’s cognitive loading was reduced to conserve their attention resources and generate less stress.

The intervention contains a novel visual strategy, namely CBV, to ameliorate this problem. CBV is able to attract the attention of adolescents with ASD, and seems to enable them to focus on the dynamic elements in a video clip. Accordingly, it can be used to help such adolescents increase their awareness and guide their understanding toward the actual meaning and emotional value of facial expressions in specific social situations. Many scholars have suggested that a relatively constrained viewing area limits the attentional frame, which helps people with ASD focus their attention on relevant stimuli and ignore irrelevant ones (Charlop-Christy and Daneshvar 2003; Sherer et al. 2001; Shipley-Benamou et al. 2002). This is why we believe that it is necessary to teach adolescents with ASD to pay attention to some social signals and ignore others. The rationale of using this type of video with adolescents with ASD is that it can simplify social stimuli, and help direct attention to the most relevant features and areas.

### Aims of this study

Adolescents with ASD inherently lack the ability to focus attention on the most important social features of a given situation or interaction, such as facial expressions, body movement, and relevant social cues; moreover, they tend to pay less attention to other people and their actions and focus their attention instead on non-social objects (Shic et al. 2011). We developed a CBV intervention learning system because we believe that a DV is too complex to effectively teach adolescents with ASD the skills involved in becoming aware of and recognizing the emotions generated by different facial expressions. Adolescents with ASD always pay more attention to inanimate objects than to faces (e.g. a vase put on a table or a clock hung on a wall); therefore, we provided a half-way point—a short video (less than 60 s) of a specific element within a static frame of surrounding elements (a Microsoft Research Cliplet)—to guide them in increasing their emotional awareness by paying attention to the facial cues of others. This is a novel visual strategy that supports adolescents with ASD by improving their ability to attract their attention on the relevant dynamic nonverbal cues and ignore irrelevant ones. Therefore, this research used Microsoft™ Research Cliplets app (Microsoft 2012) to capture viewing material from real-life situations and create CBV materials to mix static and dynamic elements from a video clip to test its efficacy for adolescents with ASD. We focused on investigating the facial expressions extracted from video materials to determine whether our intervention helped adolescents with ASD understand social situations and judge the emotional meaning of other people’s facial expressions.

## Methods

### Participants

We recruited, through the Taiwan Autism Association, six adolescents (4 boys, 2 girls: Yu, Han, Deng, Gung, Yen, and Yuen: all pseudonyms to guarantee anonymity) with ASD as participants [mean age = 13.6 years old; age range: 12–15 years; intelligence quotient (IQ) scores: (a) full scale IQ (FIQ) = 99.66; (b) verbal IQ (VIQ) = 101.33; and (c) performance IQ (PIQ) = 105.66). The inclusion criteria required (1) a clinical diagnosis of ASD based on DSM-IV-TR criteria, (2) no other specific disabilities, (3) not taking medications for physician- or self-diagnosed illnesses, (4) no physician-diagnosed comorbidities, (5) not undergoing any other therapies at the time of the testing, and (6) an FIQ > 90 (Table 1). All participants were fluent in Mandarin Chinese and Taiwanese. The participants’ sensory abilities were within the normal range; however, their parents and special education teachers reported that they usually manifested poor social and communication skills, rarely understood other people’s emotional expressions, and usually did not respond appropriately. Data on the participants’ intelligence, sensory abilities, and social and communication skills were based on multiple information sources: parental interviews, teachers’ reports, VIQ scores (Wechsler Intelligence Scale for Children and Adolescents), and functional language and social adaptation levels (based on clinical observations or behavior and adaptation scales). All participants had a disability identification card issued by a medical institution in Taiwan and had been counseled in special education schools and institutes in Taiwan. The study protocol was approved by our university hospital’s Institutional Review Board (IRB). All procedures performed in this study were in accordance with the standards of the institutional and comparable ethical standards. All participants signed a youth consent form, and parental consent forms were obtained before the participants were enrolled in the study.

**Table 1:** Summarized demographic information of the participants

Participants	Information				
	Age	FIQ	VIQ	PIQ	Diagnosis
Yu	15	93	87	101	ASD
Han	15	108	112	106	AS
Deng	12	110	107	110	AS
Gung	13	100	103	111	AS
Yen	12	93	98	100	ASD
Yuen	15	94	101	106	ASD
Mean	13.6	99.66	101.33	105.66	

Asperger syndrome (AS)

### Developing the Cliplets-based video intervention materials

**Fig. 1:**
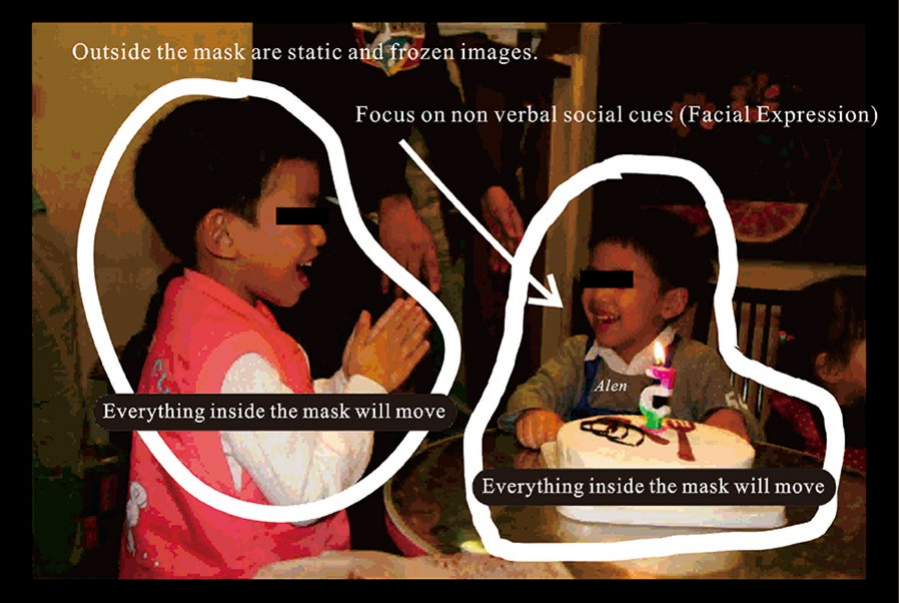
Sample of the CBV (photographic material by participant’s parents from the participant’s everyday life)

The intervention learning system takes video materials that portray everyday life activities and focuses on special moments and social clues to create the CBV, in which the desired part of the image is a movie while the incidental part is a still image (Fig. 1). The researcher created a suitable scenario and script for each CBV after discussing with each participant’s parents and special education teacher. The content of the video was based primarily on the situations that each adolescent’s age might encounter in his or her daily life at home, at school, and in the community (e.g. singing the happy birthday song for a younger brother and blowing out the candles together, or giving a present and big hug to Mother and wishing her a happy Mother’s Day). Each short script was designed to allow the participant’s parents and special education teacher to develop the stories from the adolescent’s point of view. In this manner, the CBV content was tailored for participants to learn useful social scenarios appropriate for their age and life stage. We wondered whether the improvements in their awareness of other people’s emotions would also generalize to real-life situations as there is a big difference between watching a video of a social interaction and engaging in real-life social interaction. Although the latter consists of many static and dynamic features in the interaction and in the environment, there is usually little or no chance to reflect on the situation in real life where appropriate responses are required. Therefore, we created CBV materials to give participants the chance to judge different facial expressions in real-life scenarios. By providing the adolescents with a wide range of scenarios that reflect their everyday life, and analyzing stories suitable for them, therapists might better understand what actually happens in the lives of their child clients and how the adolescents feel about it. We also included other measures, like interviews and treatment reports from parents and therapists that commented on the children’s responses to real-life situations. An effective CBV must have significant facial expressions and appropriately related body language. We focused on investigating the facial expressions and other particulars of a situation extracted from the video materials, which also was required to have rich interaction in each scenario. Highly complicated and abstract videos were excluded. Some films have many metaphorical scripts or symbolic gestures, including people using sentences such as “you are as loyal as a dog”, or “I love you more than life itself” (metaphor), and using hand gestures to form a “heart shape” to represent I love you (symbolic). This specific experimental material was avoided because most individuals with ASD have difficulty understanding abstract language, sarcasm, and metaphors (Persicke et al. 2012). In order focus on visual strategy training, we excluded the possibility of other bias interferences. In addition, we also invited therapists and special education teachers to ensure the training materials were suitable and objective to test for ASD.

### Design procedure

**Fig. 2:**
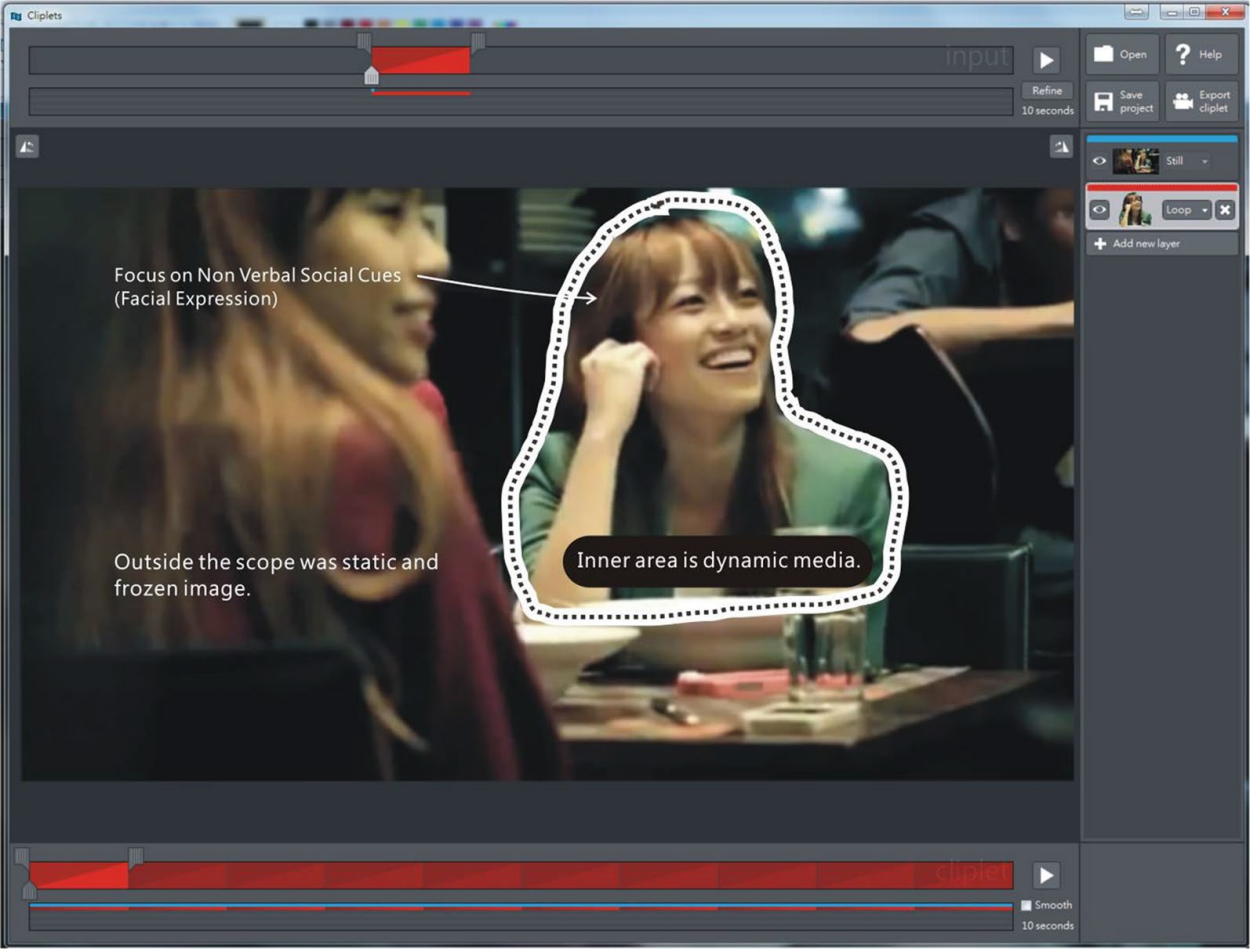
Cliplets software used to create a mixed visual medium to focus on those dynamic facial signals and ignore other contexts (Materials retrieved from Youtube: https://www.youtube.com/watch?v=zmeLEWiVreg)

**Fig. 3:**
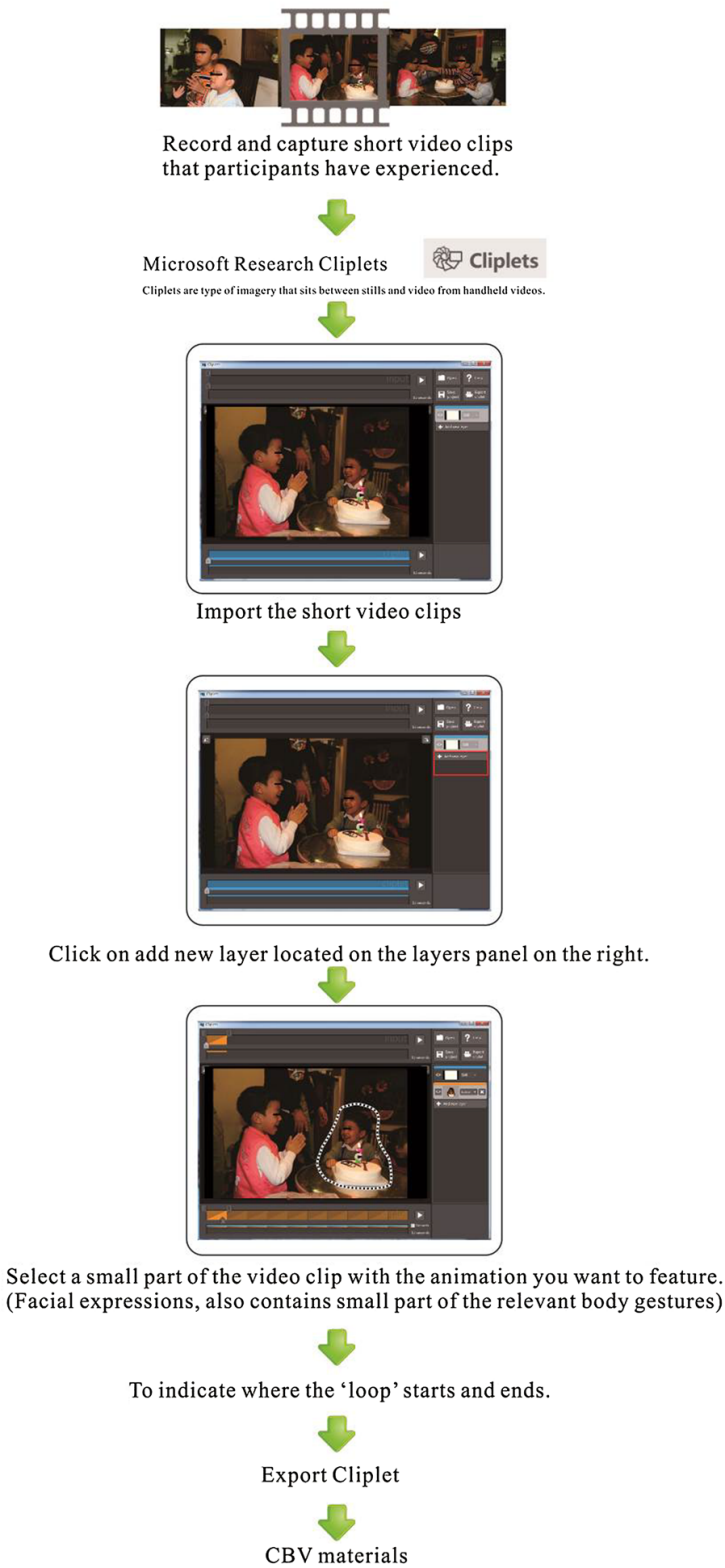
CBV materials development process

CBV materials can be used for instructing adolescents with ASD to learn how to pay attention to certain social cues. Moreover, Cliplets give the therapist a simple tool to create a mixed visual medium to direct the attention of adolescent with ASD to certain dynamic facial signals and ignore other contexts (Fig. 2). Thus, participants can focus on facial expressions and relate them to the surrounding situations. In this manner, their area of attention is constrained, thereby reducing their visual load of multiple object channels. First, we use the Cliplets app to: (a) import the short video clip of the story fragment and then choose which parts to keep as a movie and which parts to freeze. (b) We then add a new layer (a loop). (c) We next select a small part focused on the facial expressions and ensure that they are clear and easy to identify in the context of the story; therefore, the fragment we select must be an important part of the video. We want the participant to easily perceive it and try to judge it in context of the clip with the animation we want to feature and (d) decide where the loop will start and end. (e) Once that the start and end have been defined, we draw a mask around the area we want to animate (everything inside the mask will move, and everything outside the mask provides the still background image). It is also possible to create more than one animation mask by adding more than one layer, which allows two focused movement areas in the Cliplet). (f) When the CBV is finished, it can be saved in *.gif, *.mp4, or *.wmv format (Fig. 3). All CBV materials we created show various emotions and were played at the same resolution (1024 × 768) on a 27-inch display. Each video source we used was approximately 45 s long.

### Setting

We took place at three Special Education Schools in Tainan, Taiwan. All sessions were conducted in a 4 × 5-m day-treatment room at the school. Each room was equipped with chairs and a table, a personal computer with an Intel Core i7 processor, and a 27-inch LCD monitor. We selected those schools because they have special education classes and special education teachers suitable for us to conduct the test. Further, the participants selected from those schools were suitable to join the class with typically developing (TD) children to learn. Moreover, part of them received some or all of their education in regular classrooms. Those participants’ learning performance on school’s class was sufficient to meet our training the evaluations of the occupational therapist and special education teacher, and their parents also agreed to their participation in our research. We met with the participants and an accompanying occupational therapist for 1 h each week. The participants sat in front of the LCD monitor, and the researcher and occupational therapist sat in chairs beside the table. The occupational therapist recorded all of the participant’s answers during each session.

### Phases, sessions, and experimental conditions

This study used a multiple-baseline-across-participants design to analyze the experimental control in the three phases (described below). One certified occupational therapist with more than 3.5 years of experience working with adolescents with ASD conducted all sessions. She instructed all participants what to do during the intervention phase. The experiment had three phases: (a) baseline: in this phase, the researcher collected the dependent-variable data without any intervention in place; (b) intervention: the intervention learning system was used for 6–7 weeks to train the participants to notice key social clues in specific scenarios and to allow the therapist to obtain the performance data used in the assessment. During the intervention phase, the CBV training system was used to teach adolescents with ASD the skills involved in becoming aware of and recognizing the emotions generated by paying attention to the facial cues of others. This is a novel visual strategy to help focus the participants’ attention on the relevant dynamic nonverbal cues and ignore irrelevant ones. And, (c) maintenance: 4 weeks after the intervention phase, the post-training performance of the participants was assessed in order to reduce test–retest interference.

Regarding the advantages of a multiple baseline over an AB design in relation to this study, we provide some elaborations, as follows. In autism and other related fields, individual differences are quite large; therefore, therapy usually focuses on only a single individual’s development rather than a group’s. The six members of our study group have a congenital condition that manifests differently in each person; therefore, we used a single-subject in a multiple baseline across subjects design (Plavnick and Ferreri 2013) to confirm the intervention effectiveness in single subjects, despite their being ostensibly members of a group of similar subjects. This is regarded as a standard and evidence-based method in many intervention treatments used in medical, psychological, and biological research, and is especially applicable to for autism studies provided useful information for the field of special education (Kennedy and Craig 2005; Odom and Strain 2002; Wolery and Dunlap 2001). It is a fundamental experimental method for research in the field, and, in actual practice, does not require control groups or many subjects. Single-subject research has proven particularly relevant for defining educational practices at the level of the individual learner (Horner et al. 2005). Educators building individualized educational and support plans have benefited from the systematic form of experimental analysis single subject research permits (Dunlap and Kern 1997). The multiple baseline design is a style of single-subject research involving the careful measurement of multiple persons, traits, or settings both before and after a treatment. Because the manifestations of autism are different in each individual, the purposes of the research were to ascertain whether the intervention was effective and how each individual had improved (Lindgren and Doobay 2011). It has several advantages over AB designs. It is important to note that the start of the treatment conditions was staggered (started at different times) across individuals, and because of this, we could conclude that changes are due to the treatment rather than to a chance factor. By gathering data from many instances, inferences can be made about the likeliness that the measured trait generalizes to a greater population (Christ 2007).

### Baseline phase

In the baseline phase, the therapist (a) first explained to the participants the meanings of the six basic emotions (happy, sad, angry, surprise, fear, and disgust) (Ekman 2005) that they would be asked about. (b) The therapist then had the participants watch 20 standard DVs (not the CBVs) to determine the emotion described in each question. All DVs were displayed on the desktop computer. (c) After they watched the standard DVs, the participants chose one of the six facial expressions of emotion pictures according to the Facial Action Coding System (FACS) (Hamm et al. 2011) from the target emotion pictures that they thought best reflected the feelings of the characters in the videos and one of the six adjectives to answer each question. Subsequently, they tried to mimic the facial expressions in the scenarios and pretended to feel the represented emotions. In this phase, the participants’ ability to determine which emotion was expressed by which facial expression picture in the video scenario and what emotions the participants felt when they saw each facial expression picture was assessed. The answer for each situation mirrored the corresponding emotional expressions; correct and incorrect answers were identified and recorded, after which the rate of correct answers was determined. At baseline, we recorded each participant’s ability to judge the emotional meanings of the facial expressions of the characters in typical DVs.

### Intervention phase

In the intervention phase, the participants were required to watch the CBV intervention video materials to activate their understanding of the contexts and judge the questions about emotions. In the first session of the intervention phase, (a) the therapist taught the participants how to watch the CBVs on the computer and made sure that they felt comfortable using the intervention learning system. (b) Given that the intervention not only involved watching the videos, but also mimicking the facial expressions, pretending to feel the emotion, and receiving corrective feedback on the identification of the facial expressions, the CBVs were delivered as part of an intervention package. Because children with ASD tend to move around and easily become distracted, the therapist had to remind him/her to stay focused on screen and training materials. The instruction time was 40–45 min per session. (c) The participants began the experimental sessions by watching the 20 CBVs and focusing their attention on relevant social stimuli on the monitor. (d) After they watched the CBVs, the participants then selected the basic facial expression that they thought best reflected the feelings of the character in video and one of the six adjectives to answer each question. Afterwards, a therapist rated the participants’ learning performance based on these answers. When an answer was incorrect, the therapist asked the participant to watch the CBV again to observe the social stimuli in the surrounding situation, then asked the participant to determine what each social cue represented and why the appropriate facial and emotion adjective should be selected in the scenario.

### Maintenance phase

Four weeks after the intervention phase ended, the maintenance phase began. Using the baseline phase procedure, the similar scenario length, and difficulty (totally 40 DVs; 20 in baseline, 20 in maintenance phase) was used in the baseline and maintenance phases respectively (we did it to ensure they have non-interference), but a different scenario was used in the intervention phase, all the test materials were judged consistent in their length and level of difficulty by special education experts and the occupational therapist. The therapist determined whether the participants had retained the skills they had taught.

In the beginning, we asked participants’ parents and special education teachers using the same visual strategy created and recorded whole untreated videos just like normal DVs seen on TV. The DVs’ scenarios were about the perception, family love, or something related to adolescents’ daily social and emotional learning (SEL) materials. Further, we selected certain DV (totally 40 DVs; 20 in baseline phase, 20 in maintenance phase) scenario topics that were similar to, but not the same story event as used in the baseline and maintenance phases to reduce their test–retest effect. We also considered the DV’s difficulty level and tried to select a level as consistent as possible with the CBVs through a pilot study for similar aged adolescents and consulted the therapist. Relatively, in the intervention phase, we also created 20 DVs have similar topic according social and emotional interaction scenarios but not the same story in the intervention phase without used as our draft of training materials, and then those draft DVs were selected and some parts frozen (surrounding inanimate objects). The social cues, such as our facial expressions, gestures and interactions, however, were still active within the whole video to create the CBV. The CBVs used in this phase were aimed at helping to enhance participants’ ability to focus on the important parts and learn to judge different facial emotions.

### Intervention materials

In this study, we created 20 scenarios in 20 different CBVs. All six participants watched the same materials at each session to ensure test consistency. Each CBV test lasted 2 min, and each session lasted 40–45 min. All CBVs were created following the same rules, which allowed the researcher to ensure the length and difficulty of each CBV was consistent. Each CBV was then discussed with their therapist and special education teacher using the same visual strategy and questions. The scenario content was about the facial expressions of emotions that frequently occur in each participant’s daily life. The content was intended primarily to depict the six basic emotions, and the scenarios and scripts selected were approved by the 5 special educational experts and the 3 occupational therapists. The scenario stories used for the intervention phase were different from those used for the baseline and maintenance phases. Two questions were asked per story (Q1: six target emotion pictures that they thought well reflected the feelings of the characters; Q2: six adjectives to answer each question), and the participants were not prompted for answers. The questions for each CBV test used the nonverbal cues in the film to judge the participants’ emotional awareness and whether the intervention system training had increased their ability to understand the emotional expressions on other people’s faces (in the sense that the participants must understand the social behavior in the scenario and match them to the emotions expressed in the CBVs). We purposely promoted the social cues and scenario plots to make them more easily prompt the emotional expression cues for the participants to notice.

### Reliability of the data

The researcher who examined the procedural reliability of this study was the same certified occupational therapist who conducted all of the tests. In addition, we held expert meetings and conducted pilot testing to verify the test items. We followed the related experimental methods used in other studies (Castelli 2005; Chen et al. 2015) to train and test for the subjects’ ability to identify the six core emotions of happy, sad, angry, surprised, fear, and disgust (Ekman 2005), which are always difficult for adolescents with ASD to grasp and understand. We set the workflow checklist in the test procedure to follow standard operating procedures for a therapist to ensure consistency in the processes and related controls (including the video content, duration, test questions, facial expressions, case criteria, and test environment).Tithe data show that this study achieved a procedural reliability of 99 %. We also used the same visual strategy and design to control the consistency of each video material, to ensure that there were no unclear or emotionally confusing parts. We also did a pretest using TD adolescents of the same age without ASD to confirm reliability and validity of the test items. Their answers were checked by 3 therapists and 5 experts who tested for normative answers, and after the participants completed each test in each phase, we used questionnaires and interviews for expert assessment, and parental reports related to the results of the tests to ensure that social reliability and validity simulated real life. A five-point Likert scale (1—deterioration, 2—no change, 3—a little improvement, 4—fairly good improvement, 5—great improvement) was used to assess the teaching effects. The average score was 86 out of a total of 100, showing the very positive attitudes of parents and 3 therapists towards the validity of this study. In the interviews, it was also found that both the child’s parents and 3 therapists believed there were significant improvements in the child’s emotional judgement skills after the intervention, and thus they felt that it had very good teaching effects.

### Data analysis

The percentage of non-overlapping data (PND) was applied. In addition, the Kolmogorov–Smirnov test (KS-test) is a nonparametric test of the equality of continuous, one-dimensional probability distributions that can be used to compare a sample with a reference probability distribution (Goodman 1954). The KS-test attempts to determine if two datasets differ significantly, and has the advantage of making no assumption about the distribution of the data (Ziegel 2000). It is widely used to analyze the distribution patterns of data sets, especially for self-distribution differences in small samples of individuals themselves (Lilliefors 1967). Further, in our literature review, many similar studies adopted the KS-test for their research evaluation and suggested that the KS-test indeed can indicate whether the learning curve is significant or not (Chang et al. 2011; Shih et al. 2014). Moreover, the KS-test enabled us to view the data graphically which can help better understand how the data is distributed.

## Results

### Descriptive

**Fig. 4:**
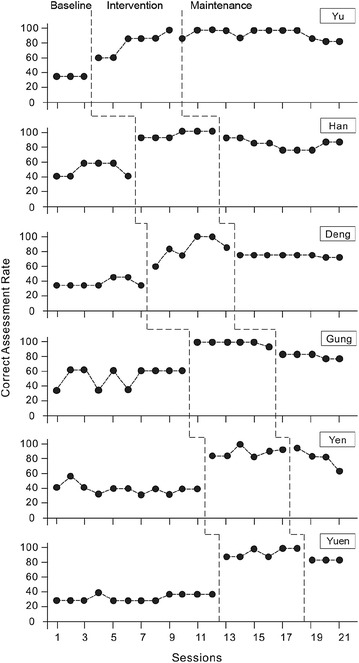
Correct assessment rates of participants’ correct responses to questions about the feelings of others during the three study phases

Experimental data on the six participants (Yu, Han, Deng, Gung, Yen, and Yuen) in each phase were analyzed. Descriptive statistics of the baseline, intervention and maintenance measures are shown in Table 2. The baseline phase consisted of 3 sessions for Yu, 6 for Han, 7 for Deng, 10 for Gung, 11 for Yen, and 12 for Yuen; however, the intervention phase consisted of 6 sessions for all participants. And lastly, the maintenance phase consisted of 12 sessions for Yu, 9 for Han, 8 for Deng, 5 for Gung, 4 for Yen, and 3 for Yuen (Fig. 4). After the participants had watched the CBVs and answered the questions during the maintenance phase, their skills in judging the emotions of others’ facial expressions were evaluated. Results showed that they had retained the skills they acquired during the maintenance phase.

**Table 2:** Summarized results of the participants

Participants	Correct rate		
	Baseline (%)	Intervention (%)	Maintenance (%)
Yu	35.71	72.92	83.18
Han	50.00	96.43	85.71
Deng	37.75	86.01	77.68
Gung	50.71	91.97	77.14
Yen	42.21	83.63	76.79
Yuen	31.55	83.04	75.00
Mean	41.32	85.67	79.25

### Intervention effect

During the baseline phase, the mean correct assessment rate was 35.71 % for Yu; however, in the intervention phase, that rate rose to 72.92 %, and then to 83.18 % in the maintenance phase. The mean correct assessment rate for Han was 50.00 % during the baseline phase. It increased to 96.43 % during the intervention phase, and was 85.71 % during the maintenance phase. During the baseline phase, the mean correct assessment rate was 37.75 % for Deng. During the intervention phase, that rate increased to 86.01 %. During the maintenance phase, it was 77.68 %. The mean correct assessment rate for Gung was 50.71 % during the baseline phase. It increased to 91.97 % during the intervention phase, and was 77.14 % during the maintenance phase. The mean correct assessment rate for Yen was 42.21 % during the baseline phase. It increased to 83.63 % during the intervention phase, and was 76.79 % during the maintenance phase. The mean correct assessment rate for Yuen was 31.55 % during the baseline phase. It increased to 83.04 % during the intervention phase, and was 75.00 % during the maintenance phase (Table 2).

The proportion of non-overlapping to total number of intervention points was calculated. All intervention points among the six participants exceeded their highest baseline point (non-overlapping), indicating highly effective training. Additionally, the Kolmogorov–Smirnov (Siegel and Castellan 1998) test curves indicate that the correct assessment rates of all the participants were significantly (*p* < .05) higher than baseline during the intervention and maintenance phases.

## Discussion

We found that the intervention system was effective in helping the six participants better judge the six basic facial expressions of others. Furthermore, for make sure more close daily status description than only test score reports. We collected data on the participants’ learning performance were based on multiple information sources: parental interviews, therapist and teachers’ reports, tests scores to ensure close to the real situation feedback. In the baseline phase, the therapist reported that the participants always paid attention to irrelevant parts of the video scenes, such as a cat lying on a chair. They frequently focused on only what interested them, for example, a picture hanging on a wall or decorative objects on a table. Moreover, via the therapist’s discussion with the participants of the character’s facial emotions, we found that they could not understand the plot events and found it difficult to recognize the emotional states of the characters. Although the participants could choose the correct words to describe the emotions, they could not identify the facial expressions that corresponded to those emotions. This could be reasons why all 6 children started with low scores (range 31.55–50.71 %) during the baseline phase.

During the intervention phase, however, using the half-static and half-dynamic video as learning materials guided them to attract their attention on the dynamic elements in the video clip. Although sometimes they still fixated on irrelevant parts of the whole video scenes, CBVs generally helped drive their attention to focus on the picture’s meanings of nonverbal feelings in facial expressions in specific social situations. They were attracted by the characters and began to ask the therapist a series of questions about why the characters’ facial expressions changed, about their gestures, and about the related social activities.

Compared to the traditional DV strategy such as VM, the CBV materials support more specific and dynamic strategy to allow the therapist to directly judge which part is needing enhancement and which can be ignore. Adolescents with ASD have chances to compare whole dynamic and part dynamic video’s differences, which can help them to imagine this video’s original situation and also link the emotional awareness with this movement. Moreover, our strategy extends the DV’s unique benefits of television/video methodology to include: fun, reduced stress, teaching flexibility, multi-sensory teaching and increased ability to gain and hold the student’s attention as well as the ability to have complete control over the observed stimuli (McCoy and Hermansen 2007). CBVs also provide more visual interaction with our participants, and attract them to discuss the video’s scene by knowing which part is important and which is not.

In addition, the therapist also found many interesting ideas during the test phase. In the baseline phase, the therapist reported that the participants always paid attention to the movie’s beginning and end, but ignored the pivotal social signals in facial expressions. The participants could not easily determine, and they frequently confused, the emotions that the six facial expressions represented. Adolescents with ASD are normally unable to judge what people are feeling based solely on facial expressions; similarly, in this study, we also found the participants always asked the therapist why some people were always sitting or standing in the corner of a room, or sitting on a chair, or always waving their hands without talking. They either do not see or do not pay attention to the facial clues. Moreover, they do not seem to understand the key points in social situations, neither their own nor those of others, and are unable to assign them an appropriate emotional status. For example, although our participants could describe what happened in the scenarios and roughly describe events that they saw occur, tell the therapist how many people were in the video, and explain why the scene was light or dark, they were unable to focus on the characters’ facial expressions. This confirms the findings of a recent study (Durham University News 2013), which says that children with ASD might be missing crucial nonverbal social cues, like facial expressions. Missing these cues generally has a negative effect on their social interaction skills and their understanding of the emotional expressions on other people’s faces. This was also mentioned in the parents’ questionnaire feedback reports about their children. They said that when their children were watching cartoons at home, they paid attention to some flashing words or to the patterns on the characters’ clothes, and that they rarely were able to discern the key elements of social signals. They spent more time searching for patterns they were interested in and ignored the important parts of the movie because they were often unable to understand the feelings of the characters.

In contrast, during the intervention phase, the intervention system trained them as to what nonverbal social cues to pay attention to in order to understand the characters’ feelings. The participants then began to ask the therapist a series of questions about why a character’s facial expressions changed, why some characters propped up their head in their hands, and why a particular character bowed his head and felt sad. These questions showed that the participants were attracted by the dynamic parts of the CBVs, had observed the moving elements, and had focused on the relevant parts. During the intervention phase, the therapists were able to interactively teach them how to observe those clues and what they meant. They eventually were able to find the social clues in the photos and compare the details of different facial clues in each scenario. Overall, the intervention system helped these six adolescents with ASD increase their comprehension of social situations and judge the emotions of others’ facial expressions. In their subsequent reports, the parents said that their children showed good improvement in facial expressions and emotional judgment, especially when they were watching familiar scenarios, and that they could point out the key elements, and discuss with them the facial expressions and the feelings of the characters.

### Limitations

This study has some limitations. First, our sample was relatively small because this strategy is a fairly new intervention strategy for individuals with ASD, it was difficult to recruit participants to join the study; moreover, the participants had limited time for the tests as many had activities and classes to take part in. Accordingly, it would be advantageous to recruit and enroll larger samples and extend the experiment time period to provide stronger evidence.

## Conclusion and future work

We conclude that a limited amount of information with structured and specific close-up social cues helped the participants improve their judgments about the emotional meaning of the facial expressions of others. In general, although adolescents with ASD may encounter other barriers, the visual support and structured situational characteristics of scenario videos were beneficial for their awareness and understanding of the feelings of others, and also helped them to improve their social-emotional function. We found that using the intervention system enabled them to recognize and understand the emotions in the facial expressions of others, which they had previously ignored. Thus, this study used an innovative but simple technique to improve the teaching of adolescents with ASD by making it more entertaining and effective. It triggered the children’s learning incentive and encouraged them to observe nonverbal facial expression signals, those benefits were not only used as DV function. This study successfully assessed the effectiveness of the CBV training material for emotion judgment by using a multiple baseline design across the participants.

 Moreover, future studies using objective devices, such as eyes tracking device and facial action coding system, to measure correct identification of emotions from facial expressions and attention status are needed. In the future, we also want to observe how their viewing behaviors changed and whether these changes can further promote their social skills used in their daily social reciprocity behaviors over time. Finally, future research is warranted to determine how reinvent visual media to increase the recognition of emotion in adolescents and other age-groups with ASD.
